# Mandatory pre-abortion counseling is a barrier to accessing safe abortion services

**DOI:** 10.11604/pamj.2020.35.80.22043

**Published:** 2020-03-19

**Authors:** Luchuo Engelbert Bain

**Affiliations:** 1 Centre for Population Studies and Health Promotion, Yaounde, Cameroon; 2 Athena Institute for Research on Innovation and Communication in the Health and Life Sciences, Vrije Universiteit, Amsterdam, Netherlands

**Keywords:** Abortion, counseling, mandatory, access, legal, pre-abortion

## Abstract

Empirical research showcases that pre-abortion counseling scarcely reverses the woman’s decision either to terminate a pregnancy or not. Growing evidence regarding the high levels of decisional certainty among women seeking abortions renders a careful rethink of the place of mandatory pre-abortion counseling packages. Mandatory counseling packages, when inscribed in the laws, at times contain false information that can deter women from going in for safe abortions. Mandatory waiting times indirectly label opting for an abortion as not being the right thing to do. In areas where abortion stigma from health care providers and communities remains highly prevalent, women are forced to incur extra expenses by travelling to other countries. I argue that pre-abortion counseling on opting-in grounds is ethically sound (enhances the woman’s reproductive autonomy), since most clients in need of abortions are certain on their decisions before the abortion care provider and do not regret these decisions after the process. Regrets are prone to be more prevalent in areas with high unsafe abortion practices, generally due to complications from excessive bleeding, pain, and post abortion infections. Allowing systematic mandatory pre-abortion counseling practice as the rule in a competent adult is unjustified ethically and empirically, is time consuming and presents the legality of abortions in most settings an oxymoron.

## Essay

### Introduction

There is a global decreasing trend in abortion related mortality and morbidity worldwide [1]. This however is untrue for sub-Saharan Africa, where maternal mortality rates ascribed to unsafe abortion remain stable at 13% [1]. Legislation is generally becoming liberal towards abortions, with many western countries allowing abortions on request up to 12 weeks of gestation [1-4]. Countries with restrictive laws on abortions suffer the most from unsafe abortion associated morbidity and mortality [1, 4, 5]. The abortion debate however remains hypocritical on legal grounds. For instance, in developing countries where abortion laws are more restrictive, they register the greatest number of maternal deaths related to unsafe abortions [1]. Also, in countries where abortions are legal, there is often no guarantee of getting access to an abortion, even when clients meet the legal requirements. Reports from Nepal indicate unacceptably many women presenting to health services for post unsafe abortion related complications, despite the legalization of abortions more than a decade ago [6]. Ignorance of existing legislation, conscientious objection by qualified health care providers, cost of having abortions, mandatory pre-abortion counseling and sometimes, distance from authorized health care facilities and stigma reduce access to safe abortion services [6-9]. Reports from the USA indicate many states are instituting even stricter abortion laws, but nothing has been done to increase or improve access to abortion services [10]. It is not rare to see reported cases of unsafe abortion or clients received later in pregnancy (second and third trimesters of pregnancy) though abortion remains legal [6]. For instance, this increased number of women seen later in the pregnancy course might have caused the French government to increase the legitimate gestational age for abortions from 12 to 14 weeks [2].

Efforts by governments to dissipate information with regards to the legal status of abortions are grossly insufficient. Practicing physicians, mid wives and other health care providers in the management chain are not aware of existing legislation [11-13]. Legal status of abortion is no direct translation of increased access to obtain an abortion [10, 14, 15]. For instance, even though abortion is legal in Zambia, girls and women still take significant risks to terminate unwanted pregnancies [7]. Levels of awareness about the legality of abortion and its provision remain low, especially among adolescents [6, 16]. Similar low levels of awareness reported from Nepal where abortions were legalized in 2002 [16], with only 40% of young girls aware of abortion laws and the legal status under which they can obtain abortion. Responsibility of states towards protecting the wellbeing of citizens is primordial. One could argue that since getting an abortion is increasingly being considered a human right, the state should fund abortion services as it does for other medical conditions. Not only are routine first trimester abortions relatively safe, it is paradoxical that governments would rather prefer to fund expensive post abortion care services [4, 17-20]. Failure to fund abortion care labels abortion already as not the right thing to do. This could go a long way to aggravate abortion related stigma, thus scaring women from getting the services they might really need.

### Discussion

#### Pre-abortion counseling, autonomy and informed consent

Three interrelated elements underlie the long-standing tradition of informed consent: patients must possess the capacity to make decisions about their care; their participation in these decisions must be voluntary; and they must be provided adequate and appropriate information [10]. Daniels *et al.* in a recent review of state mandated consent forms report one third of the information as being medically inaccurate [21]. Association of abortions to breast cancer or mental disease generally purported in most pre-counseling packages have been characterized as false from recent scientific research [22]. Current evidence is almost conclusive on the fact that abortions do not increase the risk of mental illness later in life [23-25]. On the other hand, it is rarely mentioned that abortions, especially first trimester abortions, when carried out appropriately are relatively very safe, compared to the risks of carrying a pregnancy to term [23]. Some reported barriers to access to safe abortion services where abortions are legal include mandatory waiting times and pre-abortion counseling, false information on pre-abortion counseling packages, labeling of safe abortions as not being the right thing to do by labeling the fetus an unborn child, as well as obligatory ultrasound visualization of the fetus in some circumstances [26-34]. Biased mandatory pre-abortion counseling information has also been reported from countries like Georgia, Latvia, Lithuania, Macedonia, Romania, Russia and Slovakia [35]. Indeed, Hoctor and Lamacková have argued that mandatory pre-abortion counseling simply labels an abortion as not the right thing to do, thus reinforcing abortion stigma [35].

#### Pre-abortion counseling and decisional certainty

The value of mandatory pre-abortion counseling has abundantly been put to question in the literature. Abortion counseling has 3 purposes: 1) to support the woman in making a decision for her unintended pregnancy, 2) to help her implement the decision, 3) to assist her in controlling her future fertility. Findings from empirical research indicate that most women do not need a counseling session to obtain an abortion [33, 34, 36]. In a recent systematic review on the efficacy of pre-abortion counseling in reducing subsequent unwanted pregnancies, Stewart *et al.* did not find any significant effect [37]. Indeed, since most women do not seem to want or need pre-termination counselling, therefore policies aimed at promoting mandatory counselling, would be contrary to women's wishes [33, 34]. The high levels of decisional certainty challenge the narrative that abortion decision making is exceptional compared to other healthcare decisions and requires additional protection such as: laws mandating compulsory waiting periods, counseling and ultrasound viewing. Without undermining the enormous gains accrued to pro-abortion counseling, *opting-in* in this process not only further renders more meaningful the envisaged autonomous decision of the client, but reinforces trust, respect and informed decision making. Elsewhere, mandatory waiting time, despite being a rule in many western countries offering legal abortions on request, has been described by women as not helpful [38]. Pregnancy, especially when unintended could be accompanied by some degree of stress. Some clients would therefore justly require some degree of support. Quality counseling on opting in grounds renders a trusting space, for a reasonable exchange to occur between the pregnant woman and the health care professionals, a pre-condition to make certain informed decisions. Making abortion counseling mandatory with a standard counseling package could be considered as unethical, as the women's free choice is not respected, and the very uniqueness and peculiar needs of each pregnant women might be difficult to be properly addressed.

Abortion care might not be different from other medical conditions. Gawron and Watson in a recent qualitative study conclude that women seeking abortions should be treated as moral decision-makers and given the same respect as patients making decisions about other medical procedures [39]. A non-judgmental counseling process could positively affect women, actually clarifying their concerns with regards to sensitive decisions like having an abortion. This only becomes truly ethical (autonomy enhancing and non-paternalistic) on the premise that pre-abortion counseling is voluntary. In Belgium, pre-abortion counseling is mandatory [40]. Vandamme *et al.* in Belgium have reported high levels of satisfaction amongst women using pre-abortion services [41]. This is of course difficult to ascertain since the counseling sessions are mandatory and women really have no choice. Adult women have a right to take autonomous decisions regarding their health. Mandating pre-abortion counseling before taking their decisions into consideration could breach the right to self-determination and their reproductive autonomy. Pre-abortion counseling could even be more dangerous in settings where conscientious objection for health care providers is not regulated by law. In countries where abortion service providers are limited, this could be a key issue of concern. Falling into the hands of a conscientious objector increases the risk of a biased counseling session towards dissuasion and failure to obtain an abortion. Conscientious objection does not only limit the woman's right to having an abortion, but also results in negative consequences regarding women's access to sexual and reproductive health services [42, 43]. Holistic abortion care could be a unique opportunity for women to receive a complete reproductive health counselling package. For instance, optimal contraceptive practices have the potential of reducing the burden of abortions and unintended pregnancies by more than one third alone [44].

#### Socioeconomic implications of mandatory pre-abortion counseling and restrictive legal atmospheres in the abortion debate

Mandatory pre-abortion counseling poses a serious inequity and social justice concern, as the poor are bound to suffer from failure of obtaining abortions services, even when they are legally entitled to. For instance, in Italy where 70% of gynecologists would refuse doing abortions (legal) on ground of their conscience, the rich women move to countries like the UK to obtain abortions [45, 46]. This is injustice in forcing some women to put to birth either against their will, or at an inappropriate time. Also, it is purely an unacceptable social justice burden meted on women of the lower socioeconomic class. The more restrictive a law is, the greater the prevalence of unsafe abortion related deaths [2]. Over 8-18% of these deaths result from unsafe abortions with an estimated 47,000 women dying each year [47]. Rahman has reported reproductive autonomy to be a strong predictor of unintended pregnancy rates in Bangladesh [48]. Abada and Tenkorang have reported similar findings from the Philippines, and underscored the importance of rendering reproductive decision making more autonomous among women, as one solution to reduce unintended pregnancies among women [49]. The justification of forcing women to give birth either at the wrong time, or against their will could have serious socioeconomic implications. Within the framework of the Sustainable Development Goals (SDGs), enhancing sexual and reproductive health rights of women could play significant roles in reducing gender equality (Goal 5), allowing women to continue studies and thus have the right and opportunities to quality education (Goal 4) and consequently work and contribute to economic growth (Goal 8) [50]. There is hypocrisy in making access to abortion services as a key obstacle in guaranteeing women's reproductive health rights. This simply guarantees the fact that women will turn to clandestine abortion providers and will be forced to bear children against their will. The psychological and emotional implications of having children against one's will, both from the child's and women's side still remain understudied.

It is difficult to understand the fact that governments would prefer to spend much on post abortion care services, which are far more expensive than providing safe abortion services [50-53]. Unintended pregnancies shall continue to occur even with optimal contraceptive uptake. Not all women will opt to carry pregnancies to term. The reality of opting for abortions remains. Refuting legalization of safe abortion care simply guarantees persistence of unsafe abortions, especially among the poor. With disturbing and unacceptable abortion related deaths still registered in some countries, provision of post abortion care instead of optimal access to safe abortion services remains unjustified. Advocates for safe abortion care and access must go a step further, for reduction of safe abortion fees, which still remain unacceptable to some women that required this service. Mandatory pre-abortion counseling simply implies the client has to make a minimum of two visits to the health care facility in most instances. In Utah in the United States, extending the waiting period to 72 hours was associated with a reduction in the number of women returning for abortions [54]. This is not only time consuming, but also carries an economic cost [35]. Elsewhere, this practice might be able to lead women to go past the gestational ages that qualify them for legal abortions [35]. Indeed, some women are forced to travel to other countries to obtain second and third trimester abortions. It is possible that women who are not economically viable could turn to unsafe abortion providers in such instances. Elsewhere, others might be forced to continue the pregnancy to term against their will. The social, economic, and health implications for the mother who unwillingly continuous a pregnancy to term, as well as the resulting baby could be an interesting area for future research.

### Conclusion

Since unintended pregnancies cannot be completely eliminated, and as the demand for safe abortions is a constant reality, ethically sound counseling approaches are required. Making pre-abortion counseling mandatory, does not only go against the fundamental human rights of the woman, the right to self-determination and her reproductive autonomy, but could deter a great number of women from getting access to safe abortion services, and to turn to clandestine abortion care providers. Pre-abortion counseling undoubtedly could be helpful, but even more helpful if offered on purely selective and voluntary (opting-in) grounds. Autonomous agents evidently could make mistakes with regards to what may be good to them. Pre-abortion counseling could be helpful in this case to clarify or reinforce the agent's understanding regarding the risks involved in this process, or to address specific social, psychological and contraceptive needs. This is justified on grounds that the counseling process is purely non-directive. This is however unrealistic in the abortion world, where legal, personal, religious and diverse moral convictions are active players at different levels. Not only is mandatory pre-abortion counseling unjustified from evidence-based perspective, it is unethical and ignores the woman's fundamental human right to reproductive autonomy and self-determination. In the years to come, a new pandemic of unsafe abortion related deaths could be driven by an unjustified barrier of mandatory pre-abortion counseling. Allowing this practice as the rule in competent adults requesting abortions is unjustified, unacceptable and presents the legality of abortions an oxymoron on practical grounds. Mandatory pre-abortion counseling could constitute a key obstacle to obtaining safe abortions in the years to come. Women might either shy away from seeking abortion care, revert to “shadow”; abortion providers or present for abortions late in the pregnancy course.

## Authors’ contributions

Luchuo Engelbert Bain conceived the study, carried out the literature searches and wrote the initial draft of the paper.
